# Huge Concha Bullosa and Septal Perforation: A Case Study

**DOI:** 10.22038/IJORL.2021.55647.2917

**Published:** 2021-11

**Authors:** Shohreh Norouzi, Seyyed Mohammad Tabibzadeh, Milad Zarei, Ayeh Kiani

**Affiliations:** 1 *Department of * *Otolaryngology - Head and Neck Surgery, Imam Khomeini Hospital, Ahvaz Jundishapur University of Medical Science, Ahvaz, Iran. *

**Keywords:** Concha bullosa, Functional endoscopic sinus surgery, Septal perforation

## Abstract

**Introduction:**

Concha bullosa is a usual anatomical variation of the nose and paranasal sinuses that can compromise their anatomy and function and cause such symptoms as headache, rhinosinusitis, and nasal congestion. Septal perforation is the defect of the septum that brings nasal cavities in communication and is usually caused by trauma. No septal perforation has been reported due to concha bullosa.

**Case Report:**

The case of this report was a 15-year-old female who presented with long-term nasal obstruction due to a huge concha bullosa in her right nasal cavity which perforated nasal septum. She lacked a history of any nasal septum surgery, drug abuse, cocaine, or long-term nasal spray. Endoscopic surgery was performed and our patient’s symptoms rapidly diminished after the surgery. Negative results were obtained for tuberculosis, Wegener’s granulomatosis disease, and malignancy.

**Conclusion:**

We believe that the concha bullosa presented in this case might have caused chondrocyte apoptosis due to mechanical pressure and led to septal perforation. Although uncommon, providers should be aware of the possibility of septal perforation in cases with concha bullosa when planning to perform surgery on patients with similar pathology.

## Introduction

Concha bullosa (CB) is a usual anatomical variation of the nose and paranasal sinuses in which one or both of the turbinates are inflated. The cause of this pneumatization is still unbeknown (1,2). The most common anatomical variant of the middle turbinate is concha bullosa (3). Concha bullosa is generally asymptomatic; however, patients may have complaints related to over-inflation and its consequences, such as sinusitis and nasal congestion. Occasionally it is found accidentally during a radiological examination of the paranasal sinuses (1,4). 

Perforation of the nasal septum is defined as the connection between the two nasal cavities caused by the necrosis of both the mucosal perichondrium and the mucoperiosteal, followed by cartilage and/or osteonecrosis leading to the defect of the nasal septum. (5).

The most common place for the perforation of the septum is the anterior area. The posterior or superior source is about 10%. Front perforation will cause clinical symptoms, while rear perforation will not produce too many clinical symptoms (6). Although most patients with nasal septum perforation remain asymptomatic, some might present with such symptoms as pain, crusting, bleeding, and whistling sounds from the nose (7). Among the factors that cause perforation of the nasal septum are trauma, previous septal surgeries, inflammatory diseases (e.g., Wegener's granulomatosis), and excessive use of nasal sprays (8). Literature review shows that most of the nasal septum perforation is due to foregone nasal surgery, which includes the nasal septum (9).

Although concha bullosa has not been brought up as a reason for septal perforation, if it is big enough to cause pressure on the septum, it can affect its blood supply and cause ischemic septal perforation. In this article, we showed a case of a huge middle turbinate CB, manifested as an obstructive nasal mass with a perforated nasal septum.

## Case Report

**Fig 1:**
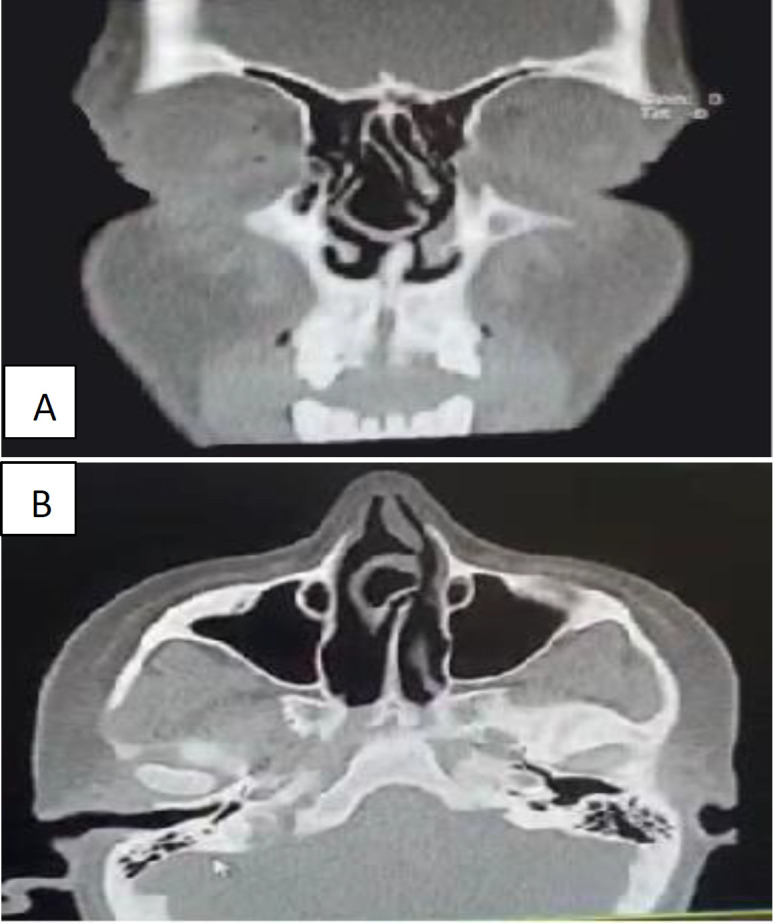
Axial (A) and coronal (B) computerized tomography scan of the paranasal sinuses in the pre-operation stage

A 15-year-old girl presented to our clinic with long-term nasal obstruction, which was bilaterally and more severe on the left side. The initial examination revealed that the patient had no history of epistaxis, trauma, headache, previous rhinoplasty, or constitutional symptoms. Moreover, the case lacked a history of any drug abuse, cocaine, and long-term nasal sprays. She also lacked any drug allergies and was healthy in other aspects, and the results of routine laboratory tests were normal. In the anterior rhinoscopy, a mass on the mucosal surface was observed, which almost completely filled the right nasal cavity. On the left side, there was a very severe nasal septal deviation that almost reached the lateral wall of the left nasal cavity. The endoscopic exam of the case showed a huge middle turbinate on the right nasal cavity that compressed the mid-portion of the nasal septum and made it deviate to the left side. Due to severe nasal septal deviation, the endoscope did not pass through the left side. No perforation was observed through the endoscopy. Since the endoscopic exam was not satisfactory, we requested a computed tomography (CT) scan of the paranasal sinuses without contrast to evaluate the whole system. Computed tomography imaging was consistent with giant CB on the right nasal cavity that passed through the nasal septum and extended somehow through the nasal septum. The septum seemed to be perforated, and there was a severe nasal septum deviation to the left side. This imaging also showed bilateral pneumatized superior concha and hypertrophic inferior conchae on the left side; nevertheless, no sinusitis was observed (Figure 1).

According to Figure 1, giant concha bullosa on the right nasal cavity passed through the nasal septum and extended through the nasal septum, and the septum perforation can be noticed.

**Fig 2:**
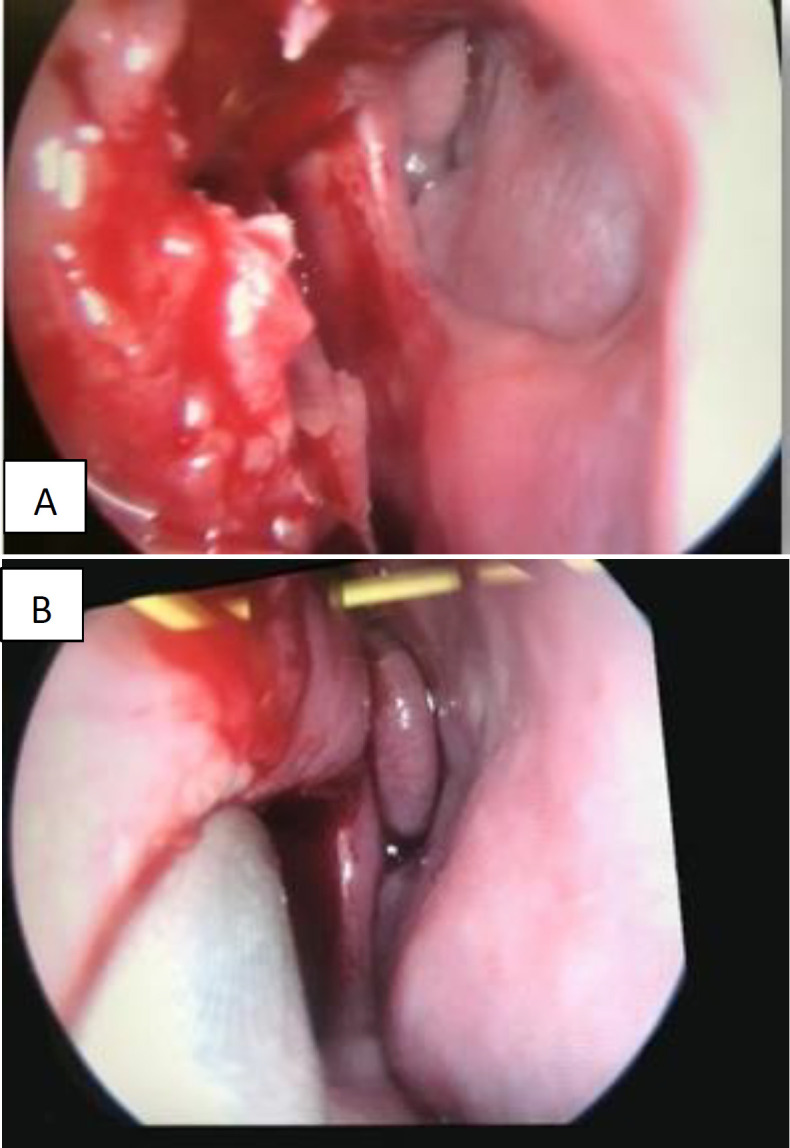
Intra-operative imaging of concha bullosa and the septal perforation in the right nasal cavity (A) and the left nasal cavity (B) after the excision of right concha bullosa

The patient was scheduled for surgery. Functional endoscopic sinus operation was performed under general anesthesia. After local anesthesia, 2 ml of 2% *lidocaine* and *epinephrine* 1: 100,000 was injected along the anterior parts of the middle turbinate. Separated the outer and inner slices of the large middle concha, and then, resect the inner slice to assess the nasal septum. The middle turbinate had pneumatized in preference to the anterior ethmoid cells. As expected, there was a circular perforation with preserved mucosa on its margins. As the pressure was reduced, we managed to perform an endoscopy on the left side (Figure 2).

Histopathological examination of the excised lesion revealed fibroconnective tissue and inflammatory cell infiltration with no evidence of granulomatous reaction or malignancy. Purified protein derivative skin test was considered negative and perinuclear anti-neutrophil cytoplasmic antibodies (p-ANCA) and classic-ANCA and angiotensin-converting enzyme levels were within normal range. Chest X-ray examination was normal. The microbial smear and culture of the specimen were negative. After the operation, the patient's symptoms alleviated rapidly.

## Discussion

The outbreak of CB varies from 14-53% (10). Computed tomography scan is a critical assessment of pneumatization of middle turbinate (8, 11). A detailed medical history, the observation of biopsy mucosal injury characteristics, and the collection of other tests, such as ANCA, can help determine the cause of septal perforation (12).

Concha bullosa is usually cured with turbo resection through nasal endoscopic surgery (13). Following any insult (chemical or physical) producing interruption of the interaction between perichondrium and cartilage, there will be an impairment of the nutrient reaches the cartilage only by the diffusion of tissue interstitial fluid. This process may eventually lead to the death of cartilage cells, which is the result of apoptosis or necrosis. When the failed interaction area is bigger, fewer nutrients pass to the cartilage and there is more chance for ischemic changes. Cartilage does not include mesenchymal stem cells; therefore, injured cartilage needs approximately no repair potential (14,15).

According to the results of studies, there is no general agreement on which technique should be preferred or which side of the bullae should be opened to improve the patient's nasal cavity and olfactory function. Braun and Stammberger suggest that removing the lateral plate has better results than removing the medial plate or crushing the middle turbinate (16). 

Based on the findings of a study conducted by Hanci and Altun (2018), the medial laminectomy should be regarded as a surgical technique for the treatment of concha bullosa (13). We preferred to perform a medial laminectomy in this case to reduce its pressure effect on the septum. We could not find a reasonable cause for septal perforation in this patient as all the investigations were negative. We believed that the concha bullosa presented in this case might cause chondrocyte apoptosis due to mechanical pressure and lead to septal perforation.

## Conclusion

We conclude that if the concha bullosa was big enough and caused pressure on the nasal septum, it could perforate the septum and cause more problematic symptoms. 

The comprehension of the pathogenesis of nasal septal perforation is momentous for practitioners who need to decide whether to recommend surgical correction or medical treatment. There seems to be a correlation between concha bullosa and septal perforation, and a surgeon should assess the patient with a thorough history, physical exam, endoscopic examination, and paranasal sinus CT inspections before the operation and provide the proper management to the patient with fewer complications.
